# Stem cell-derived secretome and dental pulp stem cells enhance sciatic nerve regeneration in a rat sciatic neurotmesis model using chitosan nerve conduits

**DOI:** 10.3389/fcell.2026.1778085

**Published:** 2026-04-10

**Authors:** Bruna Lopes, Ana Catarina Sousa, Alícia de Sousa Moreira, Patrícia Sousa, Justina Prada, Isabel Pires, Bruna Silva, Filipa João, Sandra Amado, Stefania Raimondo, Miriam Metafune, Simona Rando, Ana L. Luís, Stefano Geuna, Rui Alvites, Ana Colette Maurício

**Affiliations:** 1 Departamento de Clínicas Veterinárias, Instituto de Ciências Biomédicas de Abel Salazar (ICBAS), Universidade do Porto (UP), Porto, Portugal; 2 Centro de Estudos de Ciência Animal (CECA), Instituto de Ciências, Tecnologias e Agroambiente da Universidade do Porto (ICETA), Porto, Portugal; 3 Associate Laboratory for Animal and Veterinary Science (AL4AnimalS), Lisboa, Portugal; 4 LAQV REQUIMTE, Instituto de Ciências Biomédicas Abel Salazar, Universidade do Porto, Porto, Portugal; 5 RISE-Health, School of Medicine and Biomedical Sciences, Fernando Pessoa University, Fernando Pessoa Teaching and Culture Foundation, Gondomar, Portugal; 6 Biomedical and Health Sciences (FP-BHS), Instituto de Investigação, Inovação e Desenvolvimento Fernando Pessoa (FP-I3ID), Fundação Ensino e Cultura Fernando Pessoa, Porto, Portugal; 7 Centro de Ciência Animal e Veterinária (CECAV), Universidade de Trás-os-Montes e Alto Douro (UTAD), Vila Real, Portugal; 8 Departamento de Ciências Veterinárias, Universidade de Trás-os-Montes e Alto Douro (UTAD), Vila Real, Portugal; 9 Centre for Rapid and Sustainable Product Development (CDRSP), Polytechnic of Leiria, Marinha Grande, Portugal; 10 Associate Laboratory for Advanced Production and Intelligent Systems (ARISE), Porto, Portugal; 11 CIPER-Biomechanics and Functional Morphology Laboratory, Faculty of Human Kinetics (FMH), University of Lisbon, Lisbon, Portugal; 12 Department of Clinical and Biological Sciences, Cavalieri Ottolenghi Neuroscience Institute, University of Turin, Turin, Italy; 13 Departamento de Ciência Animal e Veterinária, Instituto Universitário de Ciências da Saúde, Cooperativa de Ensino Superior Politécnico e Universitário (IUCS-CESPU), Gandra, Portugal; 14 Campus Agrário de Vairão, Centro Clínico de Equinos de Vairão (CCEV), Vairão, Portugal

**Keywords:** conditioned medium, dental pulp stem cells, nerve conduit, peripheral nerve injury, regenerative medicine

## Abstract

**Introduction:**

Peripheral nerve injuries remain a major clinical challenge, often leading to long-term motor and sensory deficits. Stem cell-based and cell-free approaches, combined with biomaterial scaffolds, have emerged as promising strategies for nerve repair.

**Methods:**

This study evaluated the regenerative potential of human dental pulp stem cells (hDPSCs), their conditioned medium (hDPSCs-CM), and the combination of hDPSCs with olfactory mucosa mesenchymal stem cell conditioned medium (OM-MSCs-CM) in a rat model of sciatic nerve neurotmesis repaired with a chitosan-based nerve guide conduit (Reaxon®). Twenty-seven rats were allocated into experimental groups, including an uninjured control (contralateral limb), an end-to-end neurorrhaphy surgical control group, and treatment groups repaired with Reaxon® alone or combined with hDPSCs, hDPSCs-CM, or hDPSCs with OM-MSCs-CM suspended in Matrigel®. Following nerve transection, a 9–10 mm sciatic nerve gap was created. Functional recovery was monitored over 20 weeks through motor, nociceptive, behavioral, gait, stereological, histomorphometric, and electrophysiological evaluations.

**Results:**

All treatments promoted progressive motor recovery and partial restoration of nociceptive function compared to the untreated condition, although the magnitude of improvement differed among groups. The hDPSCs-CM–treated group (CMDP) showed the most favorable overall outcomes, including the lowest muscle mass loss, higher compound muscle action potential amplitudes, and functional indices approaching control values, indicating enhanced reinnervation and neuromuscular preservation. Histomorphometric and stereological analyses confirmed active regeneration across all groups, characterized by microfasciculation and thinner myelin sheaths typical of regenerating fibers.

**Discussion:**

Despite incomplete recovery, the combination of biological therapies with chitosan conduits provided an effective environment for axonal regrowth and functional improvement. These findings highlight the relevance of CMDP as a potent biological adjunct in peripheral nerve repair and support the development of cell-free, clinically translatable strategies for neuroregeneration.

## 1 Introduction

Peripheral nerve fibers are delicate and vulnerable structures, easily damaged by compression, crushing, or transection trauma (Lopes et al., 2022; Alvites et al., 2018). Peripheral nerve injuries (PNI) represent a major clinical problem, often resulting from trauma, iatrogenic procedures, or diseases (Lopes et al., 2022; Lopes et al., 2025a). These injuries are a prevalent cause of sensory and motor deficits, as they disrupt the communication between the central nervous system and the corresponding target muscles and organs. Over time, this results in behavioral alterations, impaired mobility, sensory deficits, and ultimately, permanent disabilities (Lopes et al., 2022). The wide variability regarding the type, location, severity, and complexity, makes PNI difficult to classify and treat (Alvites et al., 2018). In human peripheral somatic nerves, defects smaller than 4 cm usually have better outcomes, with axonal regeneration occurring at an approximate rate of 1–3 mm per day, depending on injury complexity and location (Alvites et al., 2018; Lopes et al., 2025b). Despite the peripheral nervous system’s intrinsic regenerative capacity, the functional recovery following extensive nerve damage remains suboptimal (Xu G. et al., 2024). So, although several medical and surgical strategies have been developed, an ideal universal treatment has not yet been established (Lopes et al., 2023). Current perspectives suggest that effective therapy will require multimodal approaches aimed at restoring motor and sensory function while preserving neuromuscular junctions to avoid neurogenic atrophy and to support reinnervation after prolonged denervation (Lopes et al., 2025b).

To facilitate the standardization of PNI, classification systems have been proposed (Alvites et al., 2018; Lopes et al., 2025b; Alvites et al., 2021). The most widely used schemes are those of Seddon and Sunderland. Seddon defined three categories: neuropraxia, axonotmesis, and neurotmesis, based on demyelination, axonal injury, and the extent of connective tissue disruption (Lopes et al., 2022; Alvites RD. et al., 2022). Sunderland further refined this model into five degrees of injury: first degree neuropraxia, fifth degree neurotmesis, and three intermediate degrees derived from axonotmesis, depending on the involvement of endoneurium, perineurium, and epineurium (Lopes et al., 2022; Alvites et al., 2020). Later, Mackinnon introduced a sixth degree to account for mixed injuries, which are probably the most common in clinical practice (Lopes et al., 2022).

Following PNI, a sequence of degeneration and regeneration occurs (Gu et al., 2024). This process involves resolution of the inflammatory response, production of neurotrophic factors, axonal regrowth, and neuronal survival (Wu et al., 2025). Schwann cells are central in the regeneration process, as they proliferate and align into Büngner bands, producing neurotrophic and adhesion molecules that guide axonal growth and remyelination (Jiang et al., 2024). However, regeneration is inherently slow, leading to prolonged denervation of target organs and muscles, often resulting in irreversible atrophy and loss of function (Talsma et al., 2024). This underlines the need for timely and effective therapeutic interventions.

In severe injuries such as neurotmesis, end-to-end (EtE) suturing remains the treatment of choice when tension-free approximation of nerve stumps is possible (Alvites et al., 2017). If not feasible, autologous nerve grafting is considered the gold standard (Lopes et al., 2022). However, it is limited by donor site morbidity, functional loss, and the challenge of obtaining grafts of adequate size and characteristics (Ni et al., 2023). To overcome the limitations associated with autologous grafts, multiple approaches have been explored to support peripheral nerve regeneration, aiming to stimulate axonal growth and improve functional recovery after PNI (Alvites et al., 2021; Shen et al., 2022; Sousa et al., 2024). Nerve guide conduits (NGCs) have been developed to direct axonal regrowth while minimizing fibrosis, and excessive inflammation (Alvites et al., 2021; Zhang and Ma, 2024). NGCs are a viable option for nerve repair, used to bridge the severed ends of the lesioned nerve, without sacrificing any healthy nerve for transplantation (Lopes et al., 2022; Lopes et al., 2023; Liu et al., 2025). Various non-degradable and biodegradable materials have been tested, the latter are preferable since they support regeneration and degrade safely without adverse local or systemic effects (Zhang and Ma, 2024; Mankavi et al., 2023). Chitosan has attracted considerable interest due to its natural origin (derived from chitin in arthropod shells and fungal cell walls), complete biodegradability, lack of toxic by-products, and bioactivity (Mawazi et al., 2024; Chicea and Nicolae-Maranciuc, 2024). Chitosan supports cell adhesion and viability, exhibits antimicrobial effects, and can be chemically or enzymatically modified for the controlled release of cytokines, antibiotics, or extracellular matrix (ECM) molecules (Lopes et al., 2025a; Gonciarz et al., 2025; Yadav et al., 2024). Preclinical studies have demonstrated that chitosan-based conduits enhance axonal regeneration, improve functional outcomes, and reduce scarring and neuroma formation (Lopes et al., 2025a; Lopes et al., 2025b; Alvites et al., 2021; Li et al., 2025; Bostani et al., 2025; Zhang et al., 2024; Ronchi et al., 2025). Reaxon® Nerve Guide (Medovent GmbH, Mainz, Germany) is a chitosan-based fully characterized and commercially available conduit (Ronchi et al., 2025). Key features include flexibility, resistance to collapse, transparency, and a positively charged luminal surface that promotes interaction with negatively charged cells and biomolecules (Gonciarz et al., 2025). These properties allow its application both for direct tubulization of nerve gaps and as a protective sheath for tension-free EtE repairs (Lopes et al., 2025a; Alvites et al., 2021; Weiss et al., 2025).

However, for long-gap nerve injuries, NGCs alone provide limited functional recovery (Redolfi et al., 2024; Stocco et al., 2023). Therefore, combining NGCs with other strategies, such as mesenchymal stem cells (MSCs) and their conditioned medium (CM) represents a promising strategy for better regeneration (Lopes et al., 2025a; Lopes et al., 2025b; Sousa et al., 2024). Among the various MSCs sources, human dental pulp stem cells (hDPSCs) have attracted particular interest because of their neurotropic properties, ease of isolation, high proliferative potential, self-renewal capacity, multilineage differentiation, and low immunogenicity (Lopes et al., 2025a; Lopes et al., 2023; Stefanska et al., 2024; Fujii et al., 2023). Their ability to differentiate into neuronal and glial lineages, combined with the release of bioactive molecules exhibiting trophic, angiogenic, and immunomodulatory effects, supports their application in neuroregenerative therapies (Lopes et al., 2025a; Mattei and Delle Monache, 2023; Tsuruta et al., 2018). Furthermore, their sustained self-renewal and cellular plasticity make them suitable for regenerative applications in PNI (Lopes et al., 2025b; Wynne et al., 2024).

The application of stem cell therapies in clinical practice is still limited by biological and technical challenges (Alvites R. et al., 2022; Sagaradze et al., 2019). Safety concerns remain, particularly the risks of immune rejection, abnormal proliferation, and inflammatory reactions, which, although uncommon due to the immunoprivileged nature of these cells, cannot be excluded. An alternative approach relies on the paracrine action of MSCs, namely the conditioned medium from human dental pulp stem cells (hDPSCs-CM) (Lopes et al., 2025b). This media contains soluble bioactive factors that can regulate inflammation, promote angiogenesis, and support nerve repair (Alvites R. et al., 2022; Ledesma-Martinez et al., 2016; Gwam et al., 2021). Similarly, olfactory mucosa mesenchymal stem cells (OM-MSCs), which originate from the neural crest and are located in the lamina propria of the nasal cavity, have attracted attention due to their versatility, chromosomal stability, and ability to maintain long-term self-renewal without donor age-related alterations (Alvites et al., 2020; Alvites et al., 2017). These cells express neural-related genes, display myogenic and neurogenic differentiation potential, and can be collected through minimally invasive procedures in both humans and small and large animal models (Alvites RD. et al., 2022; Kelly et al., 2024; Alvites et al., 2025). Importantly, their conditioned medium (OM-MSCs-CM) has been shown to stimulate glial proliferation and enhance myelination *in vitro*, highlighting its relevance for neural repair (Alvites et al., 2020; Alvites et al., 2025; Hsueh et al., 2024). Several studies indicate that the regenerative effects of MSCs are mainly mediated by their secretions (Mohd et al., 2023; Liu et al., 2024). Accordingly, hDPSCs-CM are increasingly recognized as relevant therapeutic tools for peripheral nerve injuries through paracrine mechanisms rather than direct cell replacement.

The ECM is essential for MSCs function, providing structural and biochemical support through components such as collagen, laminin, fibronectin, proteoglycans, and glycoproteins (Wang C. et al., 2024). By mediating adhesion, migration, and signaling, ECM strongly influences MSC behavior (Vakhrushev et al., 2024). Hydrogels have been used to mimic ECM properties *in vitro* and *in vivo* (Garcia-Aponte et al., 2025; Wang X. et al., 2024). Matrigel®, a basement membrane matrix derived from Engelbreth-Holm-Swarm mouse sarcoma, is particularly rich in laminin, collagen IV, heparan sulfate proteoglycans, entactin/nidogen, and multiple growth factors including Epidermal Growth Factor (EGF), Insulin-like Growth Factor (IGF), Fibroblast Growth Factor (FGF) e Transforming Growth Factor Beta (TGF-β) and tissue plasminogen activator (Xu S. et al., 2024; De Stefano et al., 2021; Passaniti et al., 2022). At physiological temperature, Matrigel® forms a 3D gel that provides a supportive microenvironment for cell adhesion, differentiation and regeneration (Passaniti et al., 2022; Zhao et al., 2025). It has been shown to promote neuronal attachment and differentiation *in vitro* and to support peripheral nerve repair *in vivo* (Wynne et al., 2024; Taisescu et al., 2025; Yu et al., 2021; Bae et al., 2022).

Animal models are essential tools for advancing peripheral nerve repair research, as they allow the evaluation of biomaterials, stem cells, and bioactive factors in a complex biological context that cannot be fully replicated *in vitro* (Lopes et al., 2023). The rat model is particularly well established in sciatic nerve transection studies due to its manageable size, cost-effectiveness, and the availability of standardized surgical protocols and outcome assessments (Alvites et al., 2021; Alvites RD. et al., 2022; Pinho et al., 2020). Moreover, the rat peripheral nervous system shares key anatomical and functional similarities with the human system, making it suitable for studying mechanisms of regeneration and testing novel therapeutic strategies (Lopes et al., 2023). Nevertheless, while the rat represents a crucial first step in preclinical research, further studies in larger animal models will be required to better mimic clinical conditions and facilitate translation to human applications (Alvites et al., 2025).

Given the current evidence supporting the use of MSC-based therapies combined with nerve guidance conduits and hydrogel matrices, this study aimed to evaluate the regenerative potential of different therapeutic strategies using a chitosan-based Reaxon® conduit associated with either hDPSCs, hDPSCs-conditioned medium, or a combination of hDPSCs with OM-MSCs-conditioned medium, all suspended in Matrigel®, in a rat model of sciatic nerve neurotmesis.

## 2 Results

### 2.1 hDPSCs cells, hDPSCs and OM-MSCs conditioned medium

The use of CM in peripheral nerve regeneration had already been addressed in previous studies from our group (Lopes et al., 2025a; Lopes et al., 2025b; Alvites et al., 2021; Alvites et al., 2025). Based on these data, P4 was selected for both hDPSCs and their CM production in the present *in vivo* assays (Lopes et al., 2025b). In addition to CM, hDPSCs and OM-MSCs at P4 were also applied as a direct therapeutic approach (Lopes et al., 2025b). All the cell lines included in this work (hDPSCs and OM-MSCs) had been previously characterized regarding morphology, proliferation, differentiation potential, and expression of neurogenic markers (Lopes et al., 2025a; Lopes et al., 2025b; Alvites RD. et al., 2022; Alvites et al., 2017). Furthermore, they had already been used in combination with the Reaxon® NGC in earlier experimental models, confirming their suitability for PNI applications (Lopes et al., 2025b; Alvites et al., 2021; Alvites RD. et al., 2022). The CM derived from OM-MSCs had also been tested in previous studies (Alvites et al., 2021; Alvites et al., 2020), further supporting its relevance for the current experimental design.

### 2.2 Functional assessment

#### 2.2.1 Motor performance

**FIGURE 1:**
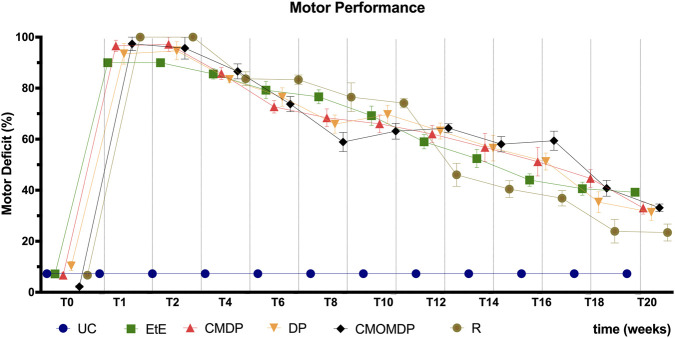
Values of motor deficit (%) over the 20 weeks of the recovery period 1 week after the injury, all groups showed high percentages of motor deficit when compared to the UC group. Deficits gradually decreased over the study weeks, and at 20 weeks, the CMOM group showed the lowest value of motor deficit. Results are presented as mean ± SEM. UC - Uninjured Control n = 27 limbs, EtE- End-to-End n = 6, R- Reaxon® n = 6, DP - hDPSCs cells n = 6, CMDP - Conditioned Medium from hDPSCs n = 6, CMOMDP - Conditioned Medium from OM-MSCs and hDPSCs cells n = 3.

The evolution of motor deficit (%) throughout the 20-week follow-up is presented in Figure 1.

Immediately after the neurotmesis procedure, all treated animals displayed a marked loss of hind limb motor function, as shown by the sharp rise in all intervened groups. At 1-week post-injury, motor deficit values remained high across all therapeutic conditions when compared to the UC group. Over the following weeks, all therapeutic groups demonstrated a gradual reduction in motor deficit, although the relative performance of each group fluctuated across intermediate timepoints. From week 10 onwards most treatment groups showed progressive functional recovery. The CMOMDP group did not exhibit a consistent trend, showing greater variability likely related to postoperative self-mutilation and consequent loss of valid samples, which compromised the reliability of its longitudinal measurements. After 20 weeks, the Reaxon® group presented the lowest percentage of motor deficit, followed by DP, CMDP, CMOMDP, and EtE. Statistical analysis revealed a significant effect of treatment group (p < 0.001). The Reaxon® group differed significantly from the EtE group (p < 0.001), while no significant differences were found among Reaxon®, DP, and CMDP. The EtE group displayed the poorest motor performance at the end of the study, showing significant differences compared with all other treated groups (p < 0.001). Despite the overall improvement across all interventions, the percentage of motor deficit remained significantly higher than in the UC group for every treated condition (p < 0.01).

#### 2.2.2 Nociceptive function

**FIGURE 2:**
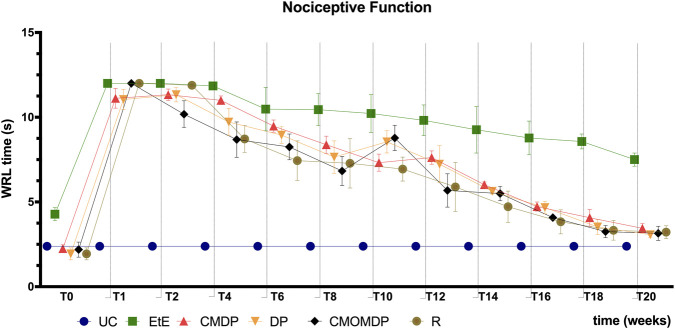
Values of Withdrawal reflex latency in seconds over 20 weeks of the recovery period. Results are presented as mean ± SEM. UC - Uninjured Control n = 27 limbs, EtE- End-to-End n = 6, R- Reaxon® n = 6, DP - hDPSCs cells n = 6, CMDP - Conditioned Medium from hDPSCs n = 6, CMOMDP - Conditioned Medium from OM-MSCs and hDPSCs cells n = 3.

The withdrawal reflex latency (WRL) values, expressed in seconds, obtained over the 20 weeks are shown in Figure 2. Following neurotmesis, all experimental groups exhibited an immediate increase in WRL time, reaching the maximum cutoff value of 12 s at the beginning, with the need to remove the paw from contact with the heating plate to avoid thermal burns. One week after neurotmesis, all experimental groups showed a marked increase in WRL, reaching the cutoff value. At this point, no statistical differences were observed among the experimental groups, but all differed significantly from UC (p < 0.0001). From week 2 onwards, a gradual reduction in WRL was observed in all treated groups, reflecting recovery of nociceptive function. The EtE group showed a slower and less pronounced decline, maintaining significantly higher WRL values than the UC group (p < 0.0001) and the other experimental groups (p < 0.0001) at week 20. In contrast, the remaining treated groups presented WRL values comparable to UC, with no significant differences between them at the end of the study.

#### 2.2.3 Walking track analysis

**FIGURE 3:**
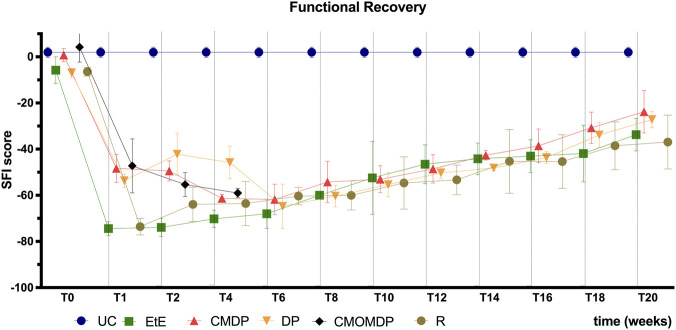
Functional assessment (SFI) over the 20-week recovery period. Results are presented as mean ± SEM. UC - Uninjured Control n = 27 limbs, EtE- End-to-End n = 6, R- Reaxon® n = 6, DP - hDPSCs cells n = 6, CMDP - Conditioned Medium from hDPSCs n = 6, CMOMDP - Conditioned Medium from OM-MSCs and hDPSCs cells n = 3.

The Static Footprint Index (SFI) values obtained over the 20 weeks are shown in Figure 3. In healthy animals, SFI values are typically close to 0, whereas values approaching −100 indicate complete sciatic nerve dysfunction. After the neurotmesis injury, all intervened groups presented severe hind limb impairment. At week 1 post-injury, despite the severe deficits observed across all conditions, the EtE group displayed the lowest SFI values. From weeks 2–4 onwards, all treated groups showed a gradual and sustained improvement in functional performance, which continued throughout the 20-week follow-up period.

At week 20, all treated groups remained significantly different from UC animals (p < 0.001). Among the treated conditions, CMDP and DP reached the highest SFI mean values, indicating the most advanced functional recovery, although no significant differences were detected between them or compared with CMOMDP. CMDP showed a significantly better outcome than R (p = 0.0436), while all other pairwise comparisons revealed no statistical significance.

**FIGURE 4:**
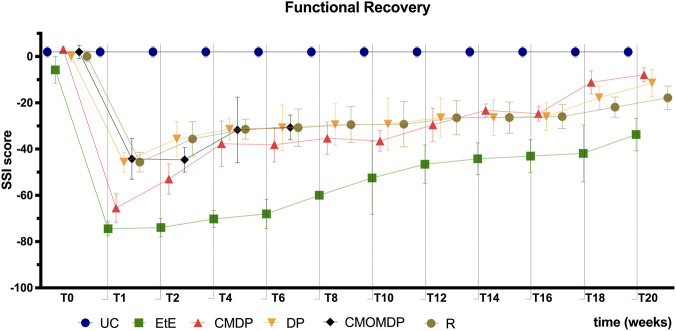
Functional assessment (SSI) during the 20-week recovery period. Results are presented as mean ± SEM. UC - Uninjured Control n = 27 limbs, EtE- End-to-End n = 6, R- Reaxon® n = 6, DP - hDPSCs cells n = 6, CMDP - Conditioned Medium from hDPSCs n = 6, CMOMDP - Conditioned Medium from OM-MSCs and hDPSCs cells n = 3.

The evolution of the static sciatic index (SSI) during the 20-week follow-up is shown in Figure 4. As expected, the UC group maintained stable SSI values close to zero throughout the experiment, consistent with normal motor function. The overall trend of SSI evolution was like SFI, supporting the consistency of the functional assessments and confirming that both video-based and photographic recording methods effectively captured locomotor performance. All operated groups exhibited a marked reduction in SSI immediately after neurotmesis, followed by a progressive but incomplete recovery over time.

Among the treated groups, EtE exhibited the lowest SSI values in the early post-injury phase and remained among the groups with poorer functional recovery throughout the study. At week 20, EtE presented the most impaired outcome, significantly different from UC (p = 0.0038), CMDP (p = 0.0089), DP (p = 0.0179), and R (p = 0.0432). In contrast, CMDP achieved the most favorable recovery, showing significant improvement over EtE (p = 0.0089) and reaching values not significantly different from UC. The DP group displayed intermediate recovery, differing significantly from EtE (p = 0.0179) and showing a trend toward normalization compared with UC (p = 0.0525). Similarly, the R group performed better than EtE (p = 0.0432) but remained significantly different from UC (p = 0.0362). The CMOMDP group also improved compared with EtE, although this difference did not reach statistical significance.

Overall, although none of the treated groups fully restored normal SSI values by week 20, CMDP and CMOMDP exhibited the better recovery profiles, clearly outperforming EtE and approaching uninjured control levels.

#### 2.2.4 Kinematic analysis

**FIGURE 5:**
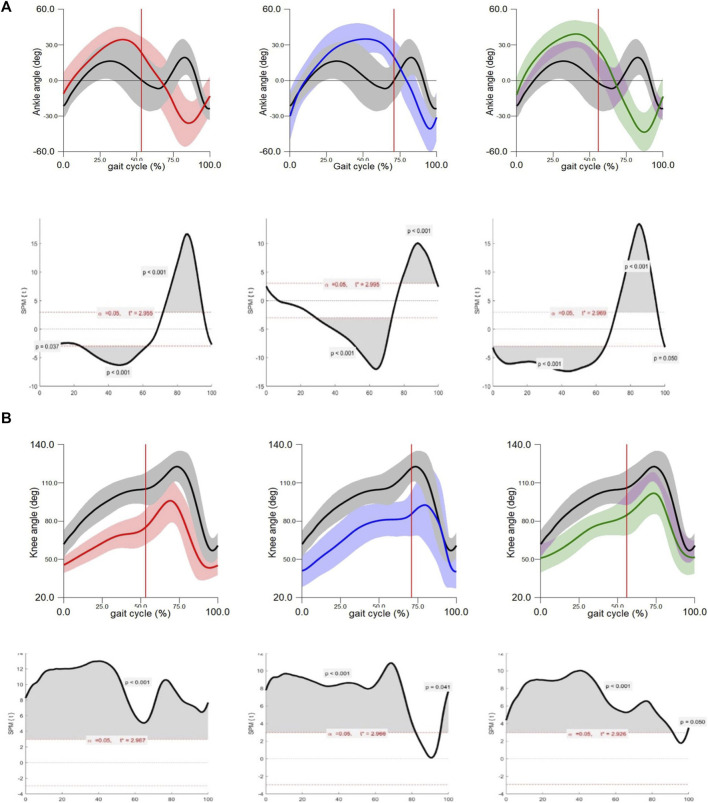
**(A)** Kinematic assessment–ankle joint angle: (red) DP; (blue) CMDP; (Green) CMOMDP; (black) control. Upper graphs show the ankle angle mean and SD, during the gait cycle. Lower graphs show SPM statistic as a function of the gait cycle. The moments of the gait cycle in which the critical threshold (t ∗) was exceeded are represented by the grey area of the lower graphs. The red vertical line corresponds to the toe-off instant. **(B)** Kinematic assessment–knee joint angle: (red) DP; (blue) CMDP; (Green) CMOMDP; (black) control. Upper graphs show the knee angle mean and SD, during the gait cycle. Lower graphs show SPM statistic as a function of the gait cycle. The moments of the gait cycle in which the critical threshold (t ∗) was exceeded are represented by the grey area of the lower graphs. The red vertical line corresponds to the toe-off instant.

The results of the kinematic assessment are shown in Figures 5A,B. The gait pattern of the ankle joint in the sagittal plane (Figure 5A) significantly differed between the three experimental groups and the control group. Groups DP and group CMDP showed an increased dorsiflexion from the midstance until the initial swing phase (p < 0.001), while group CMOMDP showed the same difference but during the entire stance phase. In the swing phase, the waveforms of the 3 experimental groups show a plantarflexion pattern, being the main difference that was found (p < 0.001). This inverted pattern, more pronounced in CMDP group, reveals that there is an inability to perform the active dorsiflexion of the ankle during swing, and the foot remains in a dropped position.

The gait pattern of the knee joint in the sagittal plane (Figure 5) significantly differed between the 3 experimental groups and the control group, during nearly the entire gait cycle, but especially during the stance phase. At initial contact, the knee is less flexed and although the pattern seems to be like the control, the maximum flexion during the swing phase is lower, which can compromise the clearance of the foot.

Regarding the spatiotemporal parameters of the gait (Table 1), all groups presented longer cycle times and less strides per minute, with CMDP group being the slowest. There was an increased stance phase (Control = 0.243 ± 0.121 s, DP = 0.414 ± 0.224 s, CMDP = 0.743 ± 0.296 s, and CMOMDP = 0.433 ± 0.186) and swing phase (Control = 0.129 ± 0.014 s, DP = 0.324 ± 0.047 s, CMDP = 0.274 ± 0.063 s, and CMOMDP = 0.326 ± 0.086 s).

**TABLE 1:** Spatiotemporal parameters (mean ± SD).

Group	DP	CMDP	CMOMDP	Control
Stance time (s)	0.414 ± 0.224	0.743 ± 0.296	0.433 ± 0.186	0.243 ± 0.121
Swing time (s)	0.324 ± 0.047	0.274 ± 0.063	0.326 ± 0.086	0.129 ± 0.014
Strides/minute	87.74 ± 21.71	62.95 ± 15.41	85.77 ± 27.48	159.26 ± 44.97
Cycle time (s)	0.738 ± 0.245	1.017 ± 0.279	0.769 ± 0.237	0.413 ± 0.142

#### 2.2.5 Electrophysiological evaluation

**FIGURE 6:**
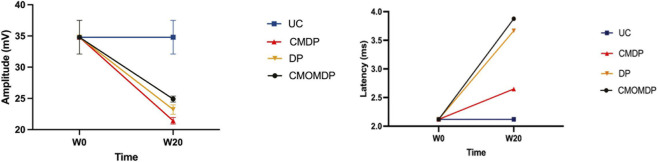
Results of amplitude and latency determined in the cranial tibial muscle at baseline (W0) and 20 weeks (W20) after sciatic nerve injury, through electrophysiological assessment. Results presented as mean ± SEM.

Electrophysiological evaluation was performed at baseline (W0) and 20 weeks (W20) after injury to assess the functional recovery of the sciatic nerve. As expected, all therapeutic groups showed markedly reduced CMAP amplitude at W0 relative to the UC group, reflecting the loss of neuromuscular connectivity following sciatic nerve injury, as in Figure 6. After 20 weeks, a clear recovery trend was observed in all treated groups, characterized by increased CMAP amplitude and decreased latency, indicative of axonal regrowth and reinnervation of the target muscle.

Among the therapeutic groups, the CMDP condition exhibited the lowest latency values, while the CMOMDP group showed the highest CMAP amplitude at W20, suggesting complementary effects in conduction velocity and muscle recruitment. Although all treated groups improved relative to W0, the DP and CMOMDP groups showed a more moderate progression. Despite these differences, all treated groups remained below the UC values, indicating that full electrophysiological recovery was not yet achieved within 20 weeks.

Overall, these results demonstrate that all therapeutic combinations supported functional nerve regeneration, with the CMDP group showing the most favorable electrophysiological profile. Although no intermediate measurements were performed, the endpoint analysis at 20 weeks provides a reliable indication of the regenerative capacity promoted by each treatment.

### 2.3 Stereological and histomorphometric analysis

#### 2.3.1 Nerve stereological analysis

**FIGURE 7:**
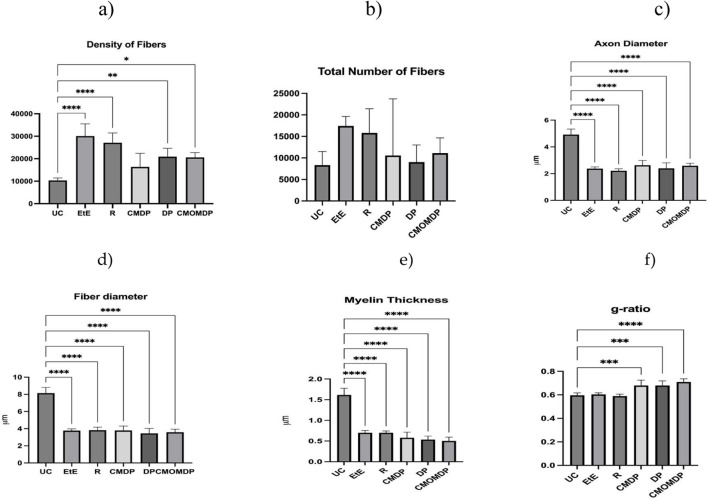
Results of the stereological assessment of sciatic nerve fibers 20 weeks after neurotmesis: **(a)** density; **(b)** total number of fibers; **(c)** axon diameter; **(d)** fiber diameter; **(e)** myelin thickness; **(f)** g-ratio. Results are presented as mean ± SEM. UC - Uninjured Control, EtE- End-to-End n = 5, R- Reaxon® n = 5, DP - hDPSCs cells n = 4, CMDP - Conditioned Medium from hDPSCs n = 5, CMOMDP - Conditioned Medium from OM-MSCs and hDPSCs cells n = 3.

**FIGURE 8:**
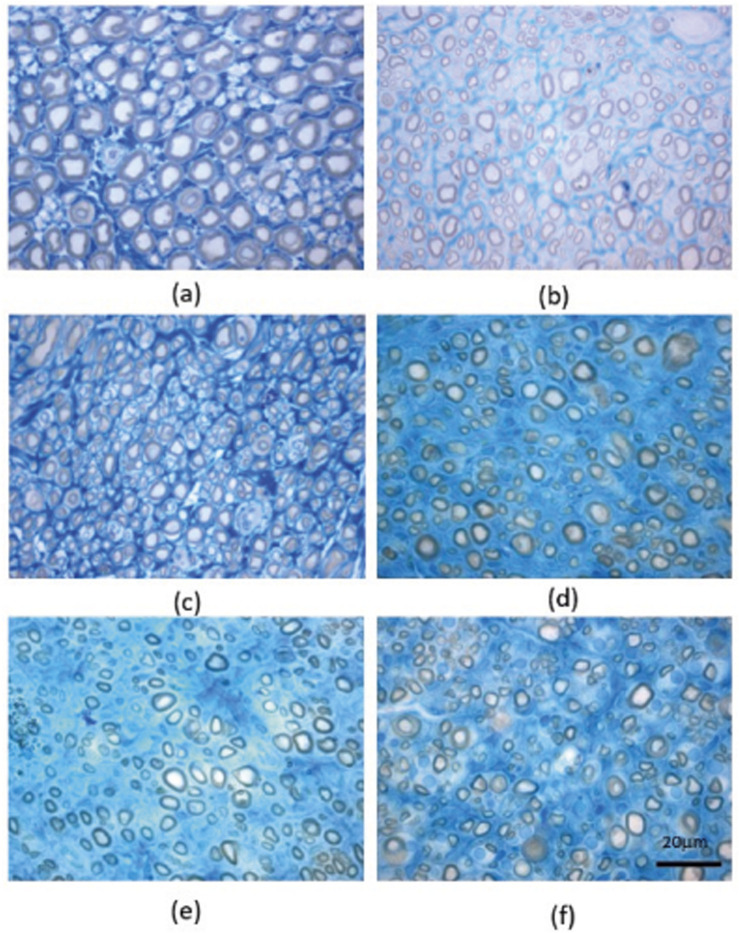
Light micrographs of toluidine blue-stained sciatic nerve semithin sections for the different groups: **(a)** UC; **(b)** EtE; **(c)** R; **(d)** CMDP; **(e)** DP; **(f)** CMOMDP. UC - Uninjured Control, EtE- End-to-End n = 5, R- Reaxon® n = 5, DP - hDPSCs cells n = 4, CMDP - Conditioned Medium from hDPSCs n = 5, CMOMDP - Conditioned Medium from OM-MSCs and hDPSCs cells n = 3.

The results of the stereological evaluation are presented in Figure 7, and representative stereological images can be found in Figure 8.

After 20 weeks of follow-up, all experimental groups exhibited morphological features consistent with active nerve regeneration. In comparison with the UC group, treated nerves displayed characteristic regenerative architecture, including microfasciculation, smaller axons and fibers, and thinner myelin sheaths. These findings were consistent between quantitative stereological data and toluidine blue-stained histological sections.

Regarding fiber density (Figure 7a), all treated groups presented higher mean values than UC (p < 0.05), reflecting axonal sprouting during regeneration. EtE and R showed slightly higher densities, although CMDP, DP, and CMOMDP reached comparable levels, and no significant differences were observed among the therapeutic groups. The total number of fibers (Figure 7b) followed the same trend, confirming ongoing regenerative activity in all conditions.

Axonal and fiber diameters (Figures 7c,d) were significantly reduced in all treated groups compared with UC (p < 0.0001), indicating the predominance of small-caliber, immature fibers typical of regenerating nerves. Within the treated cohort, EtE and R exhibited marginally larger mean diameters, whereas CMDP, DP, and CMOMDP displayed a more heterogeneous distribution, consistent with variable maturation stages but effective regeneration.

Myelin thickness (Figure 7e) was significantly lower in all experimental groups than in UC, as expected after neurotmesis. Nonetheless, CMDP and CMOMDP showed uniform myelin profiles across samples, suggesting consistent remyelination, while EtE and R presented greater variability between fascicles.

The g-ratio values (Figure 7f) remained within the physiological range (0.6–0.7) for all treated groups, with no significant intergroup differences. Slightly higher ratios in CMDP, DP, and CMOMDP indicate proportionally thinner myelin relative to axon size, compatible with ongoing maturation.

Qualitative assessment of semithin sections (Figure 8) supported these results. The UC group (Figure 8a) exhibited compact, well-organized fascicles with large, uniformly myelinated fibers. All treated groups (Figures 8b–f) displayed clear signs of regeneration, with smaller-caliber fibers and thinner sheaths. EtE and R (Figures 8b,c) showed moderate organization and microfasciculation, while CMDP and DP (Figures 8d,e) revealed a dense network of regenerating fibers with variable calibers and well-defined myelin layers. CMOMDP (Figure 8f) displayed slightly lower fiber density but maintained organized fascicular architecture and evident remyelination.

Overall, both quantitative and qualitative analyses confirm that all therapeutic strategies effectively promoted axonal regeneration and remyelination, without statistically significant superiority of any single treatment. These findings suggest that while EtE and R provide structural guidance, biologically enriched conduits such as CMDP and CMOMDP support equally robust regenerative processes, combining morphological recovery with potential trophic modulation.

#### 2.3.2 Histomorphometric muscle analysis

**FIGURE 9:**
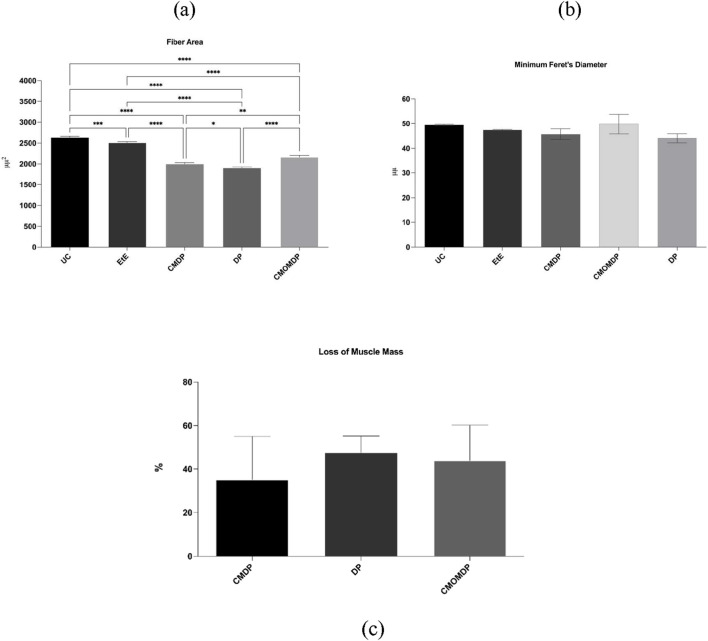
Histomorphometric analysis of the cranial tibial muscle. **(a)** Individual muscle fiber area; **(b)** Minimum Feret’s diameter of the muscle fibers; **(c)** Percentage of muscle mass loss compared with the contralateral side. Data are expressed as mean ± SEM. DP - hDPSCs cells n = 6, CMDP - Conditioned Medium from hDPSCs n = 5, CMOMDP - Conditioned Medium from OM-MSCs and hDPSCs cells n = 6.

All cranial tibial muscles associated with sciatic nerve injury exhibited reduced final weights compared with their contralateral counterparts, confirming the occurrence of neurogenic atrophy. The percentage of muscle mass loss is shown in Figure 9c. Among the therapeutic groups, CMDP presented the lowest muscle mass loss (35.7% ± 12.4%), followed by CMOMDP (41.2% ± 13.6%) and DP (46.1% ± 15.8%), indicating variable degrees of reinnervation and muscle preservation.

Histomorphometric evaluation of the cranial tibial muscles revealed statistically significant differences in muscle fiber area among groups (Figure 9a). The UC group exhibited the largest mean fiber area (2,615.4 ± 122.8 μm^2^), followed by EtE (2,468.2 ± 108.7 μm^2^). The CMDP group showed a smaller mean fiber area (1975.6 ± 96.4 μm^2^), which was significantly lower than both UC and EtE (p < 0.0001). The DP group displayed a mean value of 1,852.3 ± 88.9 μm^2^, significantly different from UC (p < 0.0001) and CMDP (p < 0.05), whereas CMOMDP presented an intermediate result (2,124.7 ± 103.2 μm^2^), differing significantly from UC (p < 0.0001) but not from CMDP.

Analysis of the minimum Feret’s diameter (Figure 9b) revealed a pattern consistent with fiber area measurements. The UC group showed the highest mean value (49.8 ± 1.3 μm), while EtE (48.9 ± 1.1 μm), CMDP (47.3 ± 1.4 μm), CMOMDP (47.9 ± 1.6 μm), and DP (46.8 ± 1.2 μm) presented slightly lower averages. No statistically significant differences were detected among the therapeutic groups.

Overall, these results confirm that all treatments partially mitigated denervation-induced muscle atrophy. Among them, CMDP demonstrated the greatest muscle mass preservation, consistent with improved reinnervation, while EtE and CMDP showed the most favorable morphometric parameters, highlighting the beneficial role of hDPSC-derived secretome in maintaining muscle trophism following peripheral nerve injury.

**FIGURE 10:**
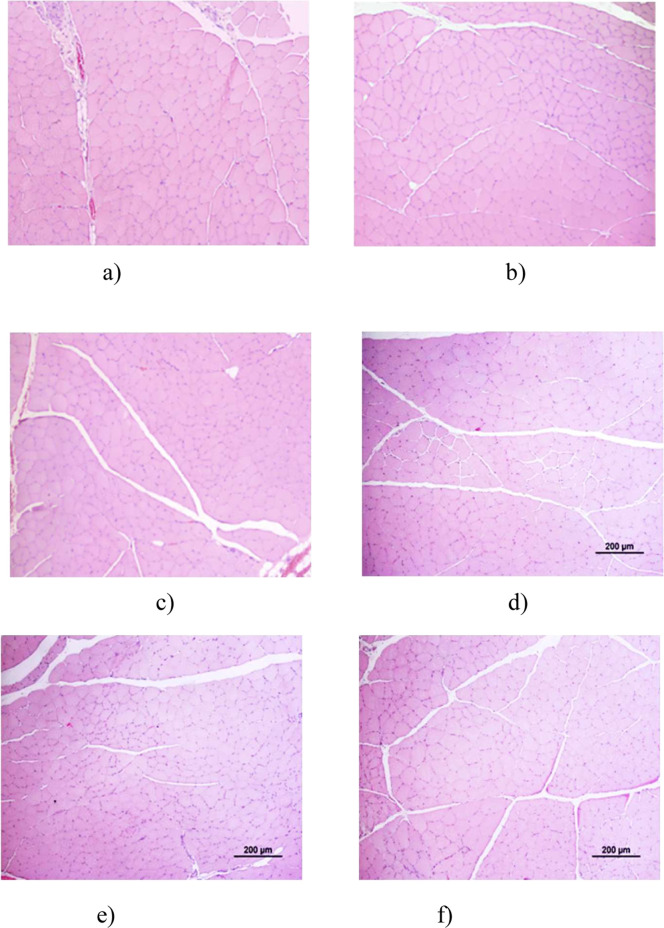
Histological images of the cranial tibial muscles subjected to histomorphometric analysis in the different groups: **(a)** UC; **(b)** EtE; **(c)** R; **(d)** CMDP; **(e)** DP; **(f)** CMOMDP. H&E, magnifications: ×100. UC - Uninjured Control, EtE- End-to-End, R- Reaxon®, DP - hDPSCs cells, CMDP - Conditioned Medium from hDPSCs, CMOMDP - Conditioned Medium from OM-MSCs and hDPSCs cells.

The qualitative histological evaluation (Figure 10) supported these quantitative findings. In the UC group (Figure 10a), muscle fibers appeared polygonal, tightly packed, and uniformly sized, reflecting normal morphology. The EtE group (Figure 10b) exhibited moderate preservation of fiber organization, with some variability in size but minimal evidence of atrophy. In contrast, the Reaxon® group (Figure 10c) showed increased endomysial spacing and occasional angular fibers, indicative of partial denervation. The CMDP group (Figure 10d) displayed more homogeneous fibers with reduced interstitial space and fewer atrophic profiles, suggesting enhanced trophic preservation and reinnervation. The DP (Figure 10e) and CMOMDP (Figure 10f) groups revealed mixed morphologies, characterized by mild fiber atrophy and occasional central nuclei, consistent with ongoing regeneration. Overall, these histological observations corroborate the morphometric results, confirming that all treatments mitigated neurogenic atrophy to varying degrees, with CMDP exhibiting the most preserved and organized muscle architecture.

## 3 Discussion

Peripheral nerve regeneration after neurotmesis relies on the combined contribution of structural guidance and biologically driven support to promote organized axonal growth and functional recovery. Traditional end-to-end repair frequently shows limited outcomes, particularly in long-gap or high-tension scenarios, motivating the development of alternatives that integrate nerve conduits with cell-derived trophic stimuli. In this study, we evaluated multiple repair strategies based on chitosan conduits and hDPSC- or OM-MSC–derived biological inputs, assessing their impact on motor, sensory, gait, structural, and electrophysiological recovery throughout a 20-week period. This integrated analysis provides a comprehensive framework for interpreting the regenerative performance of each therapeutic condition.

Throughout this work, all experimental groups subjected to sciatic nerve neurotmesis showed progressive functional and structural recovery over the 20-week period, confirming the regenerative potential of the tested therapies. The combination of chitosan-based nerve conduits and biological stimuli derived from hDPSCs and OM-MSCs proved particularly effective, supporting the gradual restoration of motor, sensory, and locomotor functions. Although complete recovery to UC levels was not achieved in any group, the global pattern of improvement, particularly in CMDP and CMOMDP, reinforces the therapeutic relevance of both structural guidance and paracrine biological modulation.

Motor function showed a progressive and sustained improvement across all treated groups, with the most pronounced gains observed after the 14th week. This gradual recovery pattern reflects the slow but continuous nature of axonal regeneration following neurotmesis, becoming functionally meaningful only once the regenerating fibers reach their distal targets (Zhang et al., 2024). Among the experimental conditions, the DP, CMDP, and Reaxon® groups displayed the most favorable motor outcomes at 20 weeks, suggesting that both the structural support provided by the chitosan conduit and the trophic influence of the hDPSC-CM and cells contributed positively to the reinnervation process. These results are consistent with previous studies from our group, where Reaxon® conduits facilitated aligned axonal growth and improved sciatic functional index recovery in similar rat models (Alvites et al., 2021). The poor results observed in the EtE group support the known limitations of direct suturing under tension, which often results in fascicular misalignment and fibrotic scarring, compromising axonal redirection and target reinnervation (Lopes et al., 2022; Zhu et al., 2022). These outcomes further strengthen the concept that combining structural and biological cues produces synergistic benefits (Lopes et al., 2025a). The Reaxon® conduit provides physical guidance and a microenvironment favorable for axonal elongation and Schwann cell migration, while the trophic factors released by hDPSCs enhance angiogenesis, modulate inflammation, and promote axonal sprouting (Pinho et al., 2020). This synergy between chitosan-based scaffolds and stem-cell-derived bioactivity has also been observed in other preclinical models (Ronchi et al., 2025; Bocker et al., 2022).

Nociceptive assessment using the WRL test confirmed the sequential pattern of sensory loss and recovery typical of sciatic neurotmesis. Immediately after injury, all experimental groups displayed complete sensory deficits, followed by a gradual recovery that became evident from week two onward. By week twenty, the DP, CMDP, and CMOMDP groups achieved WRL times close to uninjured controls, suggesting substantial restoration of nociceptive sensitivity. Conversely, the EtE group maintained significantly prolonged WRL values, consistent with incomplete small-fiber regeneration and persistent sensory impairment. These results align with previous observations from our group, in which sensory reinnervation paralleled motor function recovery in conduit-based nerve repair models (Alvites et al., 2021). These findings agree with other reports indicating that chitosan conduits promote small-fiber regeneration and that hDPSC-CM, rich in BDNF and NGF, enhance axonal elongation and glial modulation (Lopes et al., 2025a; Lopes et al., 2025b; Li et al., 2024).

Walking track analysis confirmed a progressive and consistent recovery of coordinated gait throughout the 20-week period, as evidenced by the gradual improvement of both SFI and SSI values. From week two onwards, all treated groups exhibited a steady functional gain, with CMDP and DP showing the most advanced recovery at the endpoint, achieving scores approaching those of the UC group. These findings indicate that both the direct application of hDPSCs and their CM effectively supported motor reinnervation and restoration of locomotor symmetry. The CMOMDP group, however, could not be fully assessed throughout the follow-up period due to a reduced number of valid samples. Several animals in this group exhibited self-mutilation of the digits despite all preventive measures, leading to the exclusion of affected individuals and consequently a lower sample size (n) compared with other groups. This limitation precluded the establishment of a reliable functional trajectory for CMOMDP, and the observed loss of data should not be interpreted as a treatment-related effect. In contrast, the EtE group consistently presented the lowest SFI and SSI values, confirming its limited regenerative efficiency and supporting the notion that direct suturing often leads to misalignment, fibrotic interference, and incomplete target reinnervation (Stocco et al., 2023). The clear correspondence between the behavioral indices (SFI and SSI) and kinematic gait parameters demonstrates the internal coherence of these functional measures and reinforces their value in assessing the quality and progression of recovery following PNI (Alvites et al., 2021).

Kinematic analysis provided detailed understanding of locomotor recovery, highlighting specific alterations in joint movement throughout the gait cycle. Deviations in ankle dorsiflexion and plantarflexion were particularly evident during the swing phase, confirming that this stage is the most sensitive to neuromuscular impairment after sciatic nerve injury. Animals in the CMDP and CMOMDP groups exhibited the most regular joint trajectories, with waveforms that more closely resembled those of the UC group, suggesting a more coordinated and balanced recovery pattern. In contrast, the EtE group showed pronounced asymmetries and reduced movement amplitude, consistent with incomplete reinnervation and impaired motor synchronization. These findings reinforce the functional outcomes obtained through SFI and SSI analysis, demonstrating that the combination of hDPSC-based therapies and conduit support contributes to improved dynamic gait control. The persistence of minor deviations in all treated groups indicates that, although reinnervation was functionally established, complete neuromuscular coordination and strength recovery likely require longer maturation periods (Alvites et al., 2025), and that the apparently normalized walking ability observed in animals with the naked eye is more related to compensatory and adaptive phenomena over time than to a complete and effective recovery.

Stereological evaluation confirmed that all therapeutic approaches effectively promoted axonal regeneration after neurotmesis, as shown by the presence of newly formed fascicles, smaller fibers, and thinner myelin sheaths, features typical of regenerating nerves (Alvites RD. et al., 2022; Geuna et al., 2000). Compared with the UC group, all treated groups displayed reduced axon and fiber diameters and lower myelin thickness, indicating that the regeneration process was still ongoing at 20 weeks. Despite these common features, the treatment groups, DP, CMDP and CMOMDP presented more homogeneous and organized fiber populations, suggesting that these treatments supported a more controlled and coordinated regenerative response. The slightly higher fiber density observed in the EtE and Reaxon® groups likely reflects early axonal sprouting and collateral branching, rather than advanced maturation. This interpretation aligns with previous studies describing that increased fiber density during early regeneration often corresponds to immature fibers with thin myelin and reduced conduction efficiency (Ronchi et al., 2023). Conversely, the more uniform architecture in CMDP and CMOMDP indicates better regulation of axonal growth, possibly resulting from the trophic and immunomodulatory factors contained in the hDPSC-CM and OM-MSCs, which can enhance Schwann cell activity, promote remyelination, and limit excessive sprouting (Zhang et al., 2024). The g-ratio values remained within the physiological range (0.6–0.7) in all treated groups, confirming that remyelination occurred in proportion to axonal growth. This finding is consistent with previous reports on secretome- or stem-cell-based approaches in peripheral nerve repair, which have demonstrated balanced axon–myelin development and improved structural organization compared with acellular or direct repair techniques (Raimondo et al., 2009; Ronchi et al., 2019). Qualitative observations from semithin sections further supported these quantitative findings, showing dense networks of regenerating fibers, variable calibers, and well-defined myelin layers in CMDP and DP groups, whereas EtE and Reaxon® showed greater variability and signs of delayed maturation. The morphological organization achieved with CMDP and CMOMDP suggests that biological enrichment may contribute to improved coordination between structural regeneration and functional recovery, leading to more efficient and mature nerve repair compared with conventional surgical or acellular strategies.

Muscle histomorphometry revealed that all experimental groups exhibited signs of neurogenic atrophy, confirming the persistence of denervation-induced muscle remodeling following sciatic nerve injury. However, CMDP and CMOMDP preserved significantly larger muscle fiber areas and showed lower overall mass loss compared with EtE and Reaxon®, suggesting more effective reinnervation and superior trophic support. The enhanced muscle maintenance observed in CMDP and CMOMDP is likely related to the trophic and angiogenic profile of the hDPSC-CM and OM-MSCs, known to contain growth factors such as VEGF, IGF-1 and HGF, which promote vascularization, inhibit apoptosis and support myogenic activity (Hsueh et al., 2024; Cases-Perera et al., 2022). This paracrine modulation may explain the reduced muscle atrophy and improved fiber morphology observed in these groups. Conversely, the EtE group displayed relatively larger fibers but poorer functional recovery, a paradox frequently associated with misdirected or incomplete reinnervation. This phenomenon, characterized by non-specific synaptic reconnection and impaired neuromuscular coordination, has been extensively documented in direct suture repairs under tension (Stocco et al., 2023; Kornfeld et al., 2019; Mozafari et al., 2018). Together, these findings highlight that biologically enriched conduits, particularly those incorporating stem-cell, provide a more favorable environment for restoring both nerve continuity and muscle trophism.

Electrophysiological data strongly supported the histological and functional results, demonstrating the coherence between morphological maturation and neuromuscular reactivation. At 20 weeks, CMDP exhibited high CMAP amplitudes and shorter latency values compared with some treated groups, indicating effective impulse transmission and reinnervation of the target muscles, although statistically significant superiority was not observed across all electrophysiological parameters. These electrophysiological improvements are consistent with the stereological evidence of organized fiber regeneration and uniform remyelination, suggesting that the structural maturation observed histologically translated into functional recovery. In the CMOMDP group, the addition of OM-MSC-CM may have further enhanced the regenerative microenvironment, as these cells are known to secrete bioactive molecules with neuroprotective and anti-inflammatory properties, including IL-10 and GDNF, that support Schwann cell activation and reduce fibrotic scarring (Alvites et al., 2020; Alvites et al., 2017). This dual (OM-MSCs-CM and hDPSCs) approach therefore likely provided complementary trophic support, combining the neurogenic and angiogenic effects of hDPSC with the immunomodulatory and glial-supporting actions of OM-MSCs (Stefanska et al., 2024).

Collectively, the present findings consolidate the evidence that both structural and biological strategies contribute synergistically to peripheral nerve regeneration. The use of Reaxon® conduits ensured proper orientation and limited fibrotic obstruction, while hDPSC and OM-MSCs optimized the microenvironment by providing trophic support and immunomodulation. Although CMDP demonstrated consistently favorable outcomes across functional and electrophysiological parameters, statistically significant superiority over all other biologically enriched groups was not observed in every endpoint. These findings suggest that conditioned medium represents a promising adjunct strategy, warranting further investigation (Lopes et al., 2025a).

Despite the encouraging outcomes, several limitations must be acknowledged. The relatively small sample size and inherent variability of the rat model may have limited statistical resolution for subtle intergroup differences (Lopes et al., 2023). Furthermore, kinematics and electrophysiological data were limited to endpoint assessments; longitudinal monitoring could have provided deeper insight into conduction dynamics and temporal recovery patterns. Future studies should extend the evaluation period and include molecular and transcriptomic analyses to identify specific pathways modulated by the hDPSC-CM. While the rat model offers valuable insights due to its reproducibility and accessibility, translational extrapolation to human physiology requires cautious interpretation, as nerve regeneration in humans proceeds at a considerably slower rate (Lopes et al., 2023). The next logical step is to validate these results in larger animal models, integrating quantitative electromyography and biomechanical analysis to better approximate clinical outcomes (R et al., 2021). The optimization of CM production considering donor variability, cell passage, and conditioning time should also be addressed (Lopes et al., 2025a; Lopes et al., 2025b). Additionally, combinatorial strategies using sequential or sustained delivery of secretome factors through biomaterials or microvesicles encapsulation could further enhance the regenerative microenvironment.

## 4 Materials and methods

### 4.1 Preparation of hDPSC, hDPSC and OM-MSCs conditioned medium

The hDPSCs did not required isolation as they are widely used, well studied, and their positive therapeutic outcomes are well documented. Previously our group extensively characterized these cells, confirming their nature as MSCs (Sousa et al., 2024; Machado et al., 2023; Caseiro et al., 2018; Sousa et al., 2022). A commercially available cell line hDPSCs (AllCells, LLC, Alameda, CA, United States [Cat. DP0037F, Lot no. DPSC090411-01)] was used. The cells were then expanded and cultured under standard conditions (37 °C, 5% CO_2_, and a humidified atmosphere) using a basal medium composed of αMEM, GlutaMAXTM Supplement, no nucleosides (Gibco, 32561029), which was supplemented with 10% (v/v) fetal bovine serum (FBS) (Gibco, A3160802), 100 IU/mL penicillin, 0.1 mg/mL streptomycin (Gibco, 15140122), 2.05 μg/mL amphotericin B (Gibco, 15290026), and 10 mM HEPES solution (Gibco, 15630122). For cryopreservation, cells were stored in the basal medium supplemented with 10% dimethyl sulfoxide (DMSO) (Sigma-Aldrich®) in cryovials containing at least 1 × 10^6^ cells. Prior to any assay, the cells were thawed in a 37 °C water bath, collected, centrifuged, resuspended in the basal medium, counted, cultured, and maintained under the previously mentioned standard conditions. For each experimental assay, the culture medium was discarded, cells were rinsed with PBS and then detached using 0.25% Trypsin-EDTA (Sigma-Aldrich®) through a 3-min incubation under standard conditions. Following centrifugation (1,600 rpm, 10 min) and supernatant removal, cell count, and viability were assessed using a Trypan blue exclusion assay (Invitrogen™) and an automatic cell counter (Countess II FL Automated Cell Counter, Thermo Fisher Scientific®).

Once the cell cultures reached approximately 70%–80% confluence, the medium was removed, and the culture flasks were gently washed two to three times with Dulbecco’s phosphate-buffered saline (DPBS). Following this, the flasks were washed again two to three times with the basal medium composed of αMEM, without any additional supplements. For the conditioning process, non-supplemented DMEM/F12 GlutaMAXTM (10565018, Gibco®, Thermo Fisher Scientific®, Waltham, MA, United States) was added to the flasks, which were then incubated under standard conditions. The CM, rich in factors secreted by the cells, was collected after 48 h of conditioning. The collected DPSCs-CM was concentrated five times (5×). After collection, the medium was centrifuged for 10 min at 1,600 rpm, the supernatant was collected and then filtered using a 0.2 μm syringe filter (Filtropur S, PES, Sarstedt®, Nümbrecht, Germany). For the concentration process, PierceTM Protein Concentrator, 3k MWCO, 5–20 mL tubes (88,525, Thermo Scientific®, Waltham, MA, United States) were used. Before use, the concentrators were sterilized according to the manufacturer’s instructions. Briefly, the upper chamber of each concentrator was filled with 70% ethanol (v/v) and centrifuged at 300× g for 10 min. After centrifugation, the ethanol was discarded, and the same process was repeated with DPBS. Each concentrator was subjected to two cycles of this procedure, followed by a 10-min drying period in a laminar flow hood. Finally, the upper chambers were filled with the initial CM (at 1× concentration) and underwent another round of centrifugation, repeated as needed to achieve the desired 5× concentration. The concentrated CM was stored at −20 °C and later analyzed using the Luminex™ 200 system (Luminex, Austin, TX, United States) by Eve Technologies Corp. (Calgary, Alberta) to identify specific biomarkers. Our group already studied the different components and benefits of using these cells CM (Lopes et al., 2025a; Lopes et al., 2025b; Alvites et al., 2021; Alvites et al., 2025).

### 4.2 *In vivo* assays

All animal procedures were reviewed and authorized by the Organism Responsible for Animal Welfare (ORBEA) of the Abel Salazar Institute of Biomedical Sciences (ICBAS), University of Porto (Project Nr. 459/2023/ORBEA), as well as by the Portuguese Veterinary Authorities (DGAV). The work was conducted in compliance with Directive 2010/63/EU of the European Parliament and the Portuguese legislation DL 113/2013, and followed the OECD Guidance Document on the Recognition, Assessment and Use of Clinical Signs as Humane Endpoints for Experimental Animals in Safety Evaluation (2000). Care was taken to minimize discomfort and pain, and all humane endpoints were applied to ensure animal welfare. A total of 27 male Sprague-Dawley rats (*Rattus norvegicus*), aged 8–9 weeks and weighing 250–300 g (Charles River, Barcelona, Spain), were used. Only males were selected to avoid hormonal variations related to the estrous cycle. The animals were housed under standard laboratory conditions, 2–3 per cage, in temperature- and humidity-controlled rooms with a 12 h light/dark cycle. Cages contained environmental enrichment, and normal physiological activity was allowed. Standard chow and water were provided *ad libitum*. Upon arrival, all animals underwent a 2-week acclimatization period before the experiments.

Prior to surgery, CM was prepared from hDPSCs and from OM-MSCs. In addition to the CM preparations, hDPSCs at P4 were used directly as the cellular treatment and the CM from OM-MSCs was also P4. Conditioning was carried out for 48 h, after which CM was collected and stored for later use, as previously described.

#### 4.2.1 Experimental design

A total of 27 male Sprague-Dawley rats were included in this study. All animals underwent unilateral sciatic nerve neurotmesis in the right hind limb. The contralateral left hind limb remained uninjured and was used as an internal uninjured control (UC) for comparative purposes.

Throughout the manuscript, “n” refers to the number of animals per experimental group unless otherwise specified. For the UC group, the number refers to contralateral limbs (n = 27 limbs). These contralateral limbs were used as within-animal controls and were not considered independent biological replicates.

Animals were randomly allocated into six experimental groups as follows:

- Group 1 — Uninjured Control (UC): contralateral uninjured limbs (n = 27 limbs)
- Group 2 — End-to-End neurorrhaphy (EtE): direct epineural repair (n = 6 animals)
- Group 3 — Reaxon® nerve guide conduit (R): suture of the nerve stumps into the chitosan conduit (n = 6 animals)
- Group 4 — Reaxon® + hDPSCs-conditioned medium (CMDP): administration of 150 μL of hDPSCs-CM suspended in 150 μL of Matrigel® within the conduit (n = 6 animals)
- Group 5 — Reaxon® + hDPSCs + OM-MSCs-conditioned medium (CMOMDP): administration of 1 × 10^6^ hDPSCs suspended in 150 μL OM-MSCs-CM and 150 μL of Matrigel® (n = 3 animals)
- Group 6 — Reaxon® + hDPSCs (DP): administration of 1 × 10^6^ hDPSCs suspended in 300 μL of Matrigel® (n = 6 animals)

Reaxon® nerve guide conduits measuring 2.1 mm in internal diameter and 15 mm in length were used in all conduit-based groups. Surgical transection and repair were performed in the right hind limb, leaving a 9–10 mm gap between proximal and distal nerve stumps in the conduit groups.

The primary endpoints included longitudinal assessment of motor performance, nociceptive function, behavioral recovery, and gait kinematics. Secondary endpoints included electrophysiological evaluation, stereological analysis of regenerated nerve fibers, and histomorphometric assessment of the cranial tibial muscle following tissue collection.

All experimental groups initially comprised six animals, except for the CMOMDP group (Group 5), which included three animals that completed the experimental protocol. The uninjured control group consisted of 27 contralateral limbs. Occasional missing functional data points occurred due to digit autotomy, which precluded reliable measurement; however, animals were retained in the longitudinal analyses whenever possible.

For nerve morphometric analysis, technical exclusions were applied only when samples were not analyzable due to processing artifacts (absence of the distal nerve segment, non-transverse sectioning, or severe tissue damage). Samples exhibiting absent or minimal regeneration were retained, as these represent biological outcomes rather than technical failures. After exclusions, at least three analyzable samples per experimental group were available for quantitative nerve analysis.

In muscle histomorphometric evaluation, one sample was excluded due to processing-related artifacts that prevented reliable measurement. The final number of samples included in each specific outcome measure is indicated in the corresponding figure legends.

#### 4.2.2 Surgical procedures

The anesthetic protocol used involved an intraperitoneal injection of a combination of Xylazine and Ketamine (Rompun® (Bayer Portugal®, Carnaxide, Portugal) and Imalgene® (Merial®, Boehringer Ingelheim Portugal®, Lisboa, Portugal) at a dosage of 1.25 mg/9 mg per 100 g of body weight. Once anesthesia was confirmed, the animals were positioned in left lateral recumbency, and the right limb was prepped for surgery by shaving and sterilization. The surgical procedures were performed under an M-650 operating microscope (Leica Microsystems, Wetzlar, Germany). A surgical incision was made, beginning at the greater trochanter of the femur and extending distally to the mid-thigh. The subcutaneous tissue was carefully dissected, allowing for the separation of the vastus lateralis and biceps femoris muscles, which exposed the sciatic nerve. A soft tissue retractor was used to keep the muscles apart, creating a larger working area. The sciatic nerve, once isolated from surrounding tissue, was immobilized and then fully transected using straight microsurgical scissors, inducing a neurotmesis lesion. The transection was carried out just proximal to the bifurcation of the sciatic nerve into its primary branches, the common peroneal nerve and tibial nerve. The contralateral left sciatic nerve was used as a control, forming part of the UC group. The transected nerves underwent one of several therapeutic interventions. In the EtE group, the nerve ends were aligned anatomically with minimal separation between the nerve stumps, and 2–4 simple interrupted epineural microsutures using 7/0 monofilament nylon were applied to maintain alignment and prevent rotation. In the groups using Reaxon®, approximately 3 mm of both the proximal and distal nerve stumps were placed inside Reaxon® NGCs and secured with 2–4 microsutures, also using 7/0 monofilament nylon, ensuring correct orientation. A gap of 9–10 mm was left between the nerve ends. The different cellular compositions (hDPSCs, hDPSCs-CM and OM-MSCs-CM) was injected into the NGC, filling the tube and making direct contact with the nerve injury. Following the completion of the therapeutic procedures, the muscle, subcutaneous tissue, and skin layers were closed with 4–0 absorbable simple interrupted sutures. A deterrent substance was applied to the right paw of the operated animals to prevent self-mutilation due to denervation. The animals were monitored during recovery from anesthesia and returned to their original cages and social groups once normal behavior and activity were observed. Post-surgical care included the administration of carprofen (2–5 mg/kg SC QD) for 5 days, and throughout the study, the animals were regularly assessed for signs of contractures, self-mutilation, wounds, and skin infections.

#### 4.2.3 Functional assessment

After surgery and treatment administration, animals were subjected to a set of tests to evaluate functional recovery related to nerve regeneration (Varejão ASM-P et al., 2004). Baseline measurements of functional and behavioral parameters were obtained in all animals at week 0, before the procedure of neurotmesis. Postoperative evaluations were first conducted after 1 and 2 weeks, followed by assessments every 2 weeks until week 20. All tests were carried out by the same trained operator to avoid variability between examiners. For each session, animals were chosen at random, and the operator remained blinded to their group allocation to ensure impartiality. The procedures were performed under calm conditions to minimize stress and prevent interference with the animals’ performance.

##### 4.2.3.1 Motor performance

**FIGURE 11:**
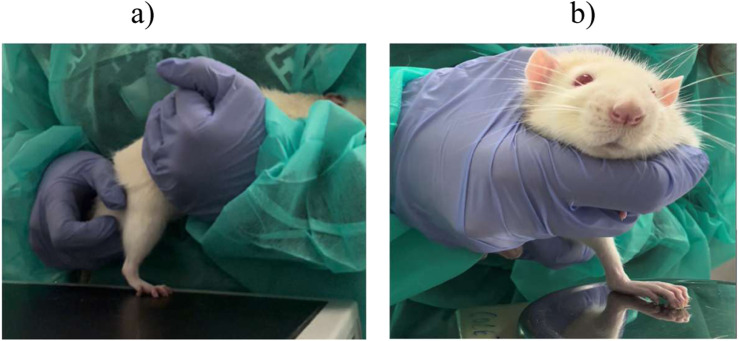
**(a)** Execution of the WRL test, with positioning of the right paw on the heating plate to test nociception. **(b)** Execution of the EPT test, with extension of the paw on the weighing machine to test motor performance.

Motor performance was assessed using the extensor postural thrust (EPT) test, as previously described (Alvites et al., 2021; Alvites RD. et al., 2022; Pinho et al., 2020; Varejão ASM-P et al., 2004). In this test, the animal is wrapped in a protective cloth, leaving the head and the limb to be tested exposed. The animal is then suspended above a digital weighing scale (model TM 560; Gibertini, Milan, Italy), and its body is gradually lowered towards it. With its head visible, the animal can visually anticipate contact with the scale, voluntarily extending the exposed limb (Figure 11). The approach is made so that the limb’s distal metatarsals and digits contact the scale. The force exerted by the limb is recorded in grams for both the injured (EEPT) and uninjured (NEPT) limbs. Each measurement is taken three times, and the average of these values is used as the result. The EEPT and NEPT values are then entered into the following equation to calculate the percentage of motor functional deficit:

$$\%\text{ Motor Deficit}=\left(\frac{NEPT-EEPT}{NEPT}\right)\times 100$$

##### 4.2.3.2 Nociceptive function

The sensory function, specifically nociceptive integrity, was evaluated through the WRL test (Alvites et al., 2021; Varejão ASM-P et al., 2004). This test is based on applying thermal stimulation to the affected limb. The animal is once again wrapped in a protective cloth, leaving the limb to be tested exposed, and suspended above a heating plate set to 56 °C (model 35-D, IITC Life Science Instruments, Woodland Hill, CA, United States). The paw of the limb is then placed on the heated surface, and the time (seconds), it takes for the animal to withdraw its paw from the painful stimulus is recorded (Alvites et al., 2021; Alvites RD. et al., 2022). This measurement is repeated three times for each limb, with a 2-min interval between trials to avoid sensitization. In healthy animals, the limb is typically withdrawn from the thermal stimulus within 4.3 s or less. For animals with severe sensory impairment, the maximum contact time with the plate is set at 12 s to prevent thermal injury, after which the operator manually removes the limb to avoid burns. During the test, it is crucial to ensure that the lateral part of the paw’s palmar surface, innervated by the terminal branches of the sciatic nerve, contacts the plate. The medial part is innervated by the saphenous nerve, a branch of the femoral nerve, and there is a possibility of collateral sprouting from this nerve to adjacent areas. Proper contact of the lateral paw is essential for obtaining reliable results. In cases of muscle atrophy or contractures, gentle manual pressure may be needed to ensure the paw’s palmar surface contacts the plate. However, care must be taken to avoid activating mechanoreceptors, which could trigger limb retraction without engaging the nociceptive reflex (Alvites et al., 2021; Alvites RD. et al., 2022; Varejão ASM-P et al., 2004).

##### 4.2.3.3 Walking track analysis

**FIGURE 12:**
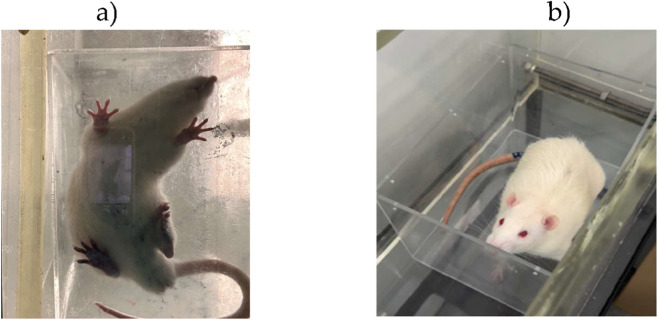
**(a)** Determination of the SFI with video recording of the animal’s paw in contact with the acrylic corridor during gait. **(a)** Analysis of the animal’s paw in contact with the acrylic corridor during gait, with determination of the PL, TS, and ITS values. **(b)** Determination of SSI with photographic record of the animal’s paw in contact with the acrylic box. EPT, extended postural thrust; WRL, withdrawal reflex latency; SFI, sciatic functional index; SSI, static sciatic index; PL, print length; TS, toe spread; ITS, intermediate toe spread.

The walking pattern was assessed by calculating the sciatic functional index (SFI). A video-based recording system was employed, adapted from conventional ink footprint methods, as previously described (Varejão ASM-P et al., 2004). Animals were placed in a transparent acrylic corridor with a video capture device positioned underneath, as in Figure 12. A shelter was installed at the end of the corridor to stimulate locomotion. Each rat was positioned at the entrance and encouraged to walk through the corridor, allowing the registration of paw prints during floor contact. For each animal, three valid steps were analyzed. A step was defined as valid when the paw outline and support points were clearly visible because of pressure on the corridor floor. Images were processed using Image-Pro Plus® 6 (Media Cybernetics, Inc.), measuring three parameters for both experimental and contralateral limbs: print length (PL), toe spread between digits 1 and 5 (TS), and intermediate toe spread between digits 2 and 4 (ITS). Mean values were incorporated into the standard SFI formulas (N stands for “normal” and E stands for “experimental”). SFI values are typically close to 0 in uninjured animals and approach −100 in cases of complete sciatic nerve transection.

$$\text{Toe Spread Factor }\left(\text{TSF}\right)=\frac{\left(\text{ETS}-\text{NTS}\right)}{\text{NTS}}$$

$$\text{Intermediate Toe Spread Factor }\left(\text{ITF}\right)=\frac{\left(\text{EIT}-\text{NIT}\right)}{\text{NIT}}$$

The calculated values are then introduced into the following formula:

$$\text{SFI}=\left(-38.3\;x\text{ PLF}\right)+\left(109.5\;x\text{ TSF}\right)+\left(13.3\;x\text{ ITF}\right)-8.8$$

Derived from the SFI, for the static sciatic index (SSI), animals were placed inside a transparent acrylic box positioned within the corridor, remaining stationary with both hind limbs in contact with the floor. Images were acquired from below, and TS and ITS were measured in triplicate for both limbs. Mean values were applied to the SSI formulas. In healthy animals, SSI values are around −5, whereas in complete sciatic nerve injury they approach −90 (Navarro, 2016).

$$\text{Toe Spread Factor }\left(\text{TSF}\right)=\frac{\left(\text{ETS}-\text{NTS}\right)}{\text{NTS}}$$

$$\text{Intermediate Toe Spread Factor }\left(\text{ITF}\right)=\frac{\left(\text{EIT}-\text{NIT}\right)}{\text{NIT}}$$

Finally, the values obtained are integrated into the following formula:

$$\text{SSI}=\left(108.44\;x\text{ TSF}\right)+\left(31.85\;x\text{ ITF}\right)\;–\;5.49$$

##### 4.2.3.4 Kinematic analysis

**FIGURE 13:**
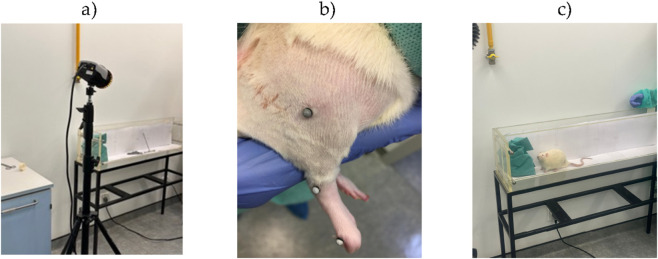
**(a)** Image capture set for one of the cameras used for the kinematic analysis. At the end of the acrylic track, a shelter is placed to attract the animal, guaranteeing a straight-line during locomotion. **(b)** Anatomical positions for placement of the reflective markers on the hind limb of the rat, with the respective ankle angle. **(c)** Animal performing the movement to record kinematic analysis with motion sensors.

Ankle kinematics during the gait cycle were recorded at the end of the 20 weeks for the three experimental groups and compared with the gait cycle pattern of the control group. Motion capture was collected through 4 infrared cameras [Qualisys Miqus 3, Qualisys AB, Sweden) (Figure 13a)], and the software Qualisys Track Manager (Qualisys, Inc.) operating at a 100 Hz framerate. Animals walked on an acrylic track with length, width, and height of, respectively, 120, 12, and 15 cm. After trichotomy, four reflective markers with 2 mm diameter were attached to 4 bony prominences on the left hind limb of the rat: the fifth metatarsal, the lateral malleolus, the lateral epicondyle and the great trochanter as in Figure 13b). All markers were placed by the same operator to avoid interindividual variations. Three body segments (foot, shank and thigh) (Figure 13c) were reconstructed using the Visual 3D software (Has-motion, Inc.), and the rat’s ankle angle and knee angle (θ°) were determined using the scalar product between the vectors representing the foot and shank (ankle angle) and the vectors representing the shank and thigh (knee angle). With this model, positive and negative values for the position of the ankle joint indicate dorsiflexion and plantarflexion respectively, and positive values for the knee joint indicate flexion. All the gait variables were normalized for 100% of the gait cycle, and for each cycle, the initial contact and the toe-off instants were identified. The normalized temporal parameters were averaged over all recorded trials, in a total of 40 gait cycles in each group.

##### 4.2.3.5 Electrophysiological evaluation

To assess the degree of reinnervation of the *tibialis cranialis muscle*, the main effector of the common peroneal branch of the sciatic nerve, electrophysiological recordings were performed at baseline (W0) and 20 weeks (W20) after sciatic nerve injury. Following the procedures described for sciatic CMAP acquisition in rodents (Pollari et al., 2018; Arn et al., 2015) evaluations were conducted without anaesthesia, using only gentle manual restraint to minimize stress and movement artefacts. Rats were positioned in dorsal recumbency on a heated surface (37 °C), and both the operated and contralateral limbs were examined.

The sciatic nerve was stimulated transcutaneously using bipolar needle stimulating electrodes inserted subcutaneously at the sciatic notch, according to validated electrode-placement methods for hindlimb CMAPs (Pollari et al., 2018). Monopolar needle recording electrodes were inserted subcutaneously over the belly of the *tibialis cranialis muscle* (active electrode) and near its distal tendon (reference), while the ground electrode was placed proximally on the flank. Recordings were acquired using a standard electromyography system. Square-wave electrical pulses of 0.1 m duration were delivered, gradually increasing stimulus intensity until the supramaximal CMAP was obtained, defined as the highest stable peak-to-peak amplitude accompanied by visible contraction of the tibialis cranialis muscle (Pollari et al., 2018; Arn et al., 2015). For each limb, latency (ms) and peak-to-peak amplitude (mV) were measured at this supramaximal intensity. Three consecutive traces were collected, and the mean value was used for analysis, as recommended for reliable CMAP quantification (Navarro, 2016).

### 4.3 Stereological and histomorphometric analysis

#### 4.3.1 Nerve stereological analysis

**FIGURE 14:**
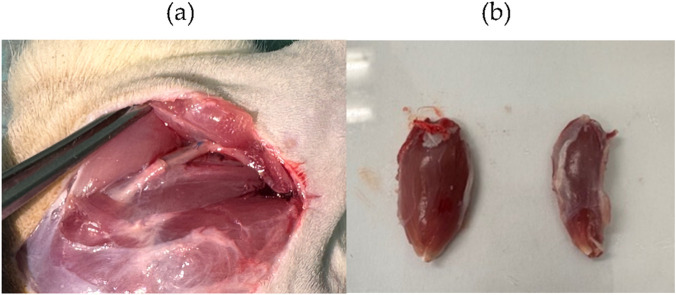
**(a)** Exposure of the sciatic nerve of the ROM therapeutic group, 20 weeks after surgery. It is possible to observe the regenerated nerve filling all the lumen of the NGC, with the two nerve tops connected. **(b)** Comparison of the mass of cranial tibial muscles of the healthy limb (left) and the one subject to neurotmesis (right) after collection at 20 weeks.

At the end of the 20-week experimental period, animals were euthanized under general anesthesia followed by a lethal intraperitoneal overdose of Eutasil® (Ceva Saúde Animal®, Algés, Portugal, 200 mg/mL, 200 mg/kg body weight). After confirmation of euthanasia, 10 mm segments of the injured sciatic nerves, distal to the lesion site, as well as uninjured control nerves, were collected and fixed for subsequent stereological evaluation by light and electron microscopy, as in Figure 14. Nerve exposure was performed using the same surgical approach as in the initial intervention. Once exposed, the sciatic nerve was covered with fixation solution (2.5% purified glutaraldehyde and 0.5% sucrose in 0.1 M Sorensen phosphate buffer, pH 7.4, 4 °C) to promote hardening and facilitate handling during excision.

Following excision, the 10 mm segment, with the proximal end properly identified, was immersed in the same fixation solution, aligned, and oriented for 5 min to avoid nerve twisting. Samples were then maintained in the fixation solution for 6–8 h, subsequently rinsed, and immersed in 1.5% sucrose wash solution in 0.1 M Sorensen phosphate buffer (pH 7.4) for 6–12 h.

Histological preparation was performed according to previously described methods (Geuna et al., 2000; Raimondo et al., 2009; Scipio FDR et al., 2008). The histomorphometric analysis included quantification of the total number of fibers (N), fiber density (N/mm^2^), axon diameter (µm), fiber diameter (µm), myelin thickness (M, µm), cross-sectional area (mm^2^), and the g-ratio (d/D).

#### 4.3.2 Histomorphometric muscle analysis

In parallel with the sciatic nerve collection, the cranial tibial muscles were excised from both injured and contralateral limbs for histomorphometric evaluation and determination of the degree of neurogenic atrophy. Muscles were weighed immediately after excision to quantify the loss of muscle mass, fixed in 4% buffered formaldehyde, and processed for routine histopathological examination using hematoxylin and eosin (H&E) staining.

From the mid-belly region of each muscle, consecutive sections of 3 µm thickness were prepared and stained. Low-magnification images (100×) were acquired using a Nikon® microscope (Nikon Corporation®, Tokyo, Japan) equipped with a Nikon® DXM1200 digital camera. The images were analyzed with ImageJ© software (Rasband, W.S., ImageJ, U. S. National Institutes of Health, Bethesda, MD, United States) using an unbiased sampling approach. For each group, individual muscle fibers were measured, and both fiber cross-sectional area and minimal Feret’s diameter (defined as the minimum distance between parallel tangents on opposite fiber borders) were determined. Measurements were obtained blindly and randomly from a minimum of 800 fibers per group by two independent and experienced operators.

### 4.4 Statistical analysis

Statistical analyses were performed using GraphPad Prism for Mac OS X (version 6.00; GraphPad Software, La Jolla, CA, United States). Data are presented as mean ± standard error of the mean (SEM), unless otherwise specified.

Normality of data distribution was assessed using the Shapiro–Wilk test. As data met the assumptions for parametric testing, group comparisons were conducted using parametric statistical methods.

Longitudinal functional outcomes, including extensor postural thrust (EPT), withdrawal reflex latency (WRL), sciatic functional index (SFI), and static sciatic index (SSI), collected repeatedly over the 20-week follow-up period, were analyzed using two-way repeated-measures ANOVA, with treatment group as the between-subject factor and time as the within-subject factor. When significant main effects or interaction effects were detected, Tukey’s multiple comparisons *post hoc* test was applied to identify pairwise differences between groups and/or timepoints.

Electrophysiological parameters (CMAP amplitude and latency), stereological measurements (fiber density, total number of fibers, axon diameter, fiber diameter, myelin thickness, cross-sectional area, and g-ratio), and muscle histomorphometric data (fiber area, minimal Feret’s diameter, and muscle mass loss) were analyzed using one-way ANOVA followed by Tukey’s *post hoc* test for multiple group comparisons.

For comparisons between injured and contralateral limbs within the same animal, paired statistical analyses were used when appropriate.

Correction for multiple comparisons was performed using Tukey’s method. A value of P < 0.05 was considered statistically significant. Statistical significance in figures is represented as follows: (*) 0.01 ≤ P < 0.05; (∗∗) 0.001 ≤ P < 0.01; (∗∗∗) 0.0001 ≤ P < 0.001 and (∗∗∗∗) P < 0.0001.

For kinematic analysis, statistical parametric mapping (SPM) was used to analyze ankle and knee joint planar angles across the normalized gait cycle. SPM unpaired t-tests were performed to compare the mean kinematic angle waveform of each experimental group with that of the control group (α = 0.05). All SPM analyses were conducted using open-source SPM1d software (version M.0.4.7, 2019.11.27) implemented in MATLAB.

## 5 Conclusion

The present study reinforces the regenerative potential of integrating structural and biological strategies for peripheral nerve repair. The use of chitosan-based NGC enriched with hDPSC and OM-MSC-CM promoted consistent functional and structural recovery after sciatic neurotmesis, as evidenced by improvements in motor, sensory, and locomotor performance throughout the 20-week follow-up. Among the tested approaches, CMDP and CMOMDP achieved the most favorable outcomes, reflecting the synergistic action of physical guidance provided by the conduit and the paracrine modulation exerted by the stem-cell secretome.

Stereological, histomorphometric, and electrophysiological analyses corroborated active axonal regeneration, remyelination, and partial reinnervation of the target muscle, consistent with the progressive functional gains observed behaviorally. These results demonstrate that cell-free secretome-based therapies can reproduce many of the beneficial effects of stem-cell transplantation, offering a safer and more standardized alternative.

Nevertheless, complete recovery to control levels was not achieved within the experimental period, indicating that longer maturation times and refined delivery strategies may be required to reach full functional restoration. Despite these limitations, the current findings support the view that biologically enriched chitosan conduits constitute a promising platform for translational nerve repair, bridging structural guidance with trophic and immunomodulatory support.

Future studies should focus on optimizing CM production and release kinetics, extending follow-up periods, and validating these results in larger animal models to better approximate clinical conditions. The cumulative evidence from this and previous work highlights the relevance of integrating functional, morphological, and electrophysiological assessments to comprehensively evaluate the efficacy of novel therapeutic combinations in peripheral nerve regeneration.

## Data Availability

The raw data supporting the conclusions of this article will be made available by the authors, without undue reservation.
